# The immune modulation effects of gemcitabine plus cisplatin induction chemotherapy in nasopharyngeal carcinoma

**DOI:** 10.1002/cam4.4705

**Published:** 2022-03-30

**Authors:** Xiao‐Min Li, Xiao‐Min Zhang, Jun‐Yan Li, Ning Jiang, Lei Chen, Ling‐Long Tang, Yan‐Ping Mao, Wen‐Fei Li, Guan‐Qun Zhou, Ying‐Qin Li, Na Liu, Yuan Zhang, Jun Ma

**Affiliations:** ^1^ State Key Laboratory of Oncology in South China, Collaborative Innovation Center of Cancer Medicine, Guangdong Key Laboratory of Nasopharyngeal Carcinoma Diagnosis and Therapy Sun Yat‐sen University Cancer Center Guangzhou China; ^2^ Jiangsu Cancer Hospital, Jiangsu Institute of Cancer Research The Affiliated Cancer Hospital of Nanjing Medical University Nanjing China

**Keywords:** cisplatin, combination therapy, gemcitabine, immune modulation, nasopharyngeal carcinoma

## Abstract

**Background:**

Studies are trying to add immunotherapy to gemcitabine and cisplatin (GP) induction chemotherapy, the standard therapy, in nasopharyngeal carcinoma (NPC) patients with locoregionally advanced disease. However, how the immune system responds to GP remains unknown.

**Method:**

We examined the dynamic changes of circulating immune cells and plasma cytokines in NPC patients administered with GP.

**Result:**

After GP administration, immunosuppressive myeloid cells, including CD11b+CD14+ monocytes, CD33+ myeloid cells, CD33+CD11+ myeloid cells, total MDSCs (CD33+CD11+HLA‐DR−/low), monocytic MDSCs, and granulocytic MDSCs decreased significantly. The regulatory T cells and B cells, two important suppressive lymphocyte subpopulations, also decreased. On the other hand, the levels of CD3+ T cells, total B cells, central memory CD4+ T cells, and pro‐inflammatory cytokines (including Interleukin [IL]‐1β, IL‐6, IL‐2, IL‐5, and IL‐8) increased significantly after GP administration. Besides, GP chemotherapy did not weaken the cytotoxic activity and proliferative capacity of T cells.

**Conclusion:**

Our results showed the immune modulation effect of GP induction chemotherapy in locoregionally advanced NPC, providing a solid basis for its combination with immunotherapy.

## Graphical abstract

Through monitoring the dynamic changes in the peripheral immune cell populations and cytokines, we demonstrated that GP chemotherapy not only significantly decreased the immunosuppressive cells but also improved the T cell immunity in NPC patients. Our study provided a new insight into the anti‐tumor mechanism of gemcitabine and cisplatin chemotherapy. Furthermore, our findings offered solid basis for the combination of GP induction chemotherapy with immunotherapy in future studies.

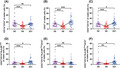

## 1 INTRODUCTION

Nasopharyngeal carcinoma (NPC), a malignancy originating from the nasopharyngeal epithelium, has an extremely unbalanced geographical and ethnic distribution. In 2018, there were about 130,000 newly diagnosed cases and 79,000 death attributed to it around the world, mainly prevalent in East and Southeast Asia.^1^, ^2^ Among newly diagnosed NPC patients, more than 70% have locoregionally advanced disease, with high risks of distant metastasis and tumor recurrence.^3^ For these patients, concurrent chemoradiotherapy with a platinum‐based agent constitutes the back‐stone of curative treatment.

In our recent published randomized trial, three cycles of gemcitabine and cisplatin (GP) induction chemotherapy before concurrent chemoradiotherapy significantly lowered the risk of disease failure and death in patients with locoregionally advanced NPC.^4^ According to this study, induction chemotherapy before concurrent chemoradiotherapy was recommended as the standard of therapy in the setting of locoregionally advanced NPC by the National Comprehensive Cancer Network, and GP was the preferred regimen.

Despite the superior therapeutic efficacy, about 15% of patients still experienced disease recurrence after the standard therapy. Currently, studies are trying to add immunotherapy, which has shown promising efficacy in many cancers including NPC,^5^, ^6^, ^7^ to GP therapy in a curative or palliative setting (NCT03984357, NCT03427827, and NCT03619824).^8^, ^9^ However, how the immune system responds to GP remains unknown, thus the basis of the above combination was lacking. In this study, we aimed to provide a comprehensive landscape of immunological changes during GP induction therapy, to suggest the combined modality with immunotherapy.

## 2 MATERIALS AND METHODS

### 2.1 Blood samples collection

The Institutional Review Board and Institutional Ethics Board of Sun Yat‐sen University Cancer Center (Guangzhou, China) approved this study (No. B2021‐023‐01). Peripheral blood was collected from healthy donors and NPC patients. In NPC patients who received GP induction chemotherapy (gemcitabine 1000 mg/m^2^, on day 1 and day 8; platinum 80 mg/m^2^ on day 1, every 21 days/cycles, for 3 cycles), peripheral blood was also collected on day 8 (before gemcitabine monotherapy) and day 21 (a day before the second cycle of treatment). Blood was collected in heparin anticoagulation tubes. And peripheral blood mononuclear cells (PBMCs) were isolated and used fresh. Written informed consent for the collection of blood samples was obtained from all subjects.

### 2.2 Flow cytometry analysis

We used flow cytometry to evaluate the phenotypic expression of PBMCs from NPC patients and healthy donors. In general, PBMCs were blocked with an FcR‐blocking reagent (Biolegend) to avoid nonspecific binding, and then stained with corresponding antibodies for 30 min at 4°C. For FOXP3 staining, cells were fixed and permeabilized with FOXP3 fix/perm buffer set from eBioscience (ThermoFisher Scientific) according to the manufacturer's protocols. Fixable Viability Dye eFluor 506 was used to distinguish between dead and living cells. Cells were run through a CYTOFLEX flow cytometer (Beckman Coulter) and results were analyzed using Flow Jo software. Antibodies used are detailed in Table S2.

### 2.3 Proliferation assay

The proliferation capacities of PBMCs from NPC patients and healthy donors were analyzed using a carboxyfluorescein diacetate succinimidyl ester cell proliferation assay (ThermoFisher Scientific) according to the protocol. The PBMCs were stimulated with IL‐2 (100 IU/mL), Muromonab‐CD3, and anti‐CD28 (1 μg/mL, Biolegend). Measurements were taken at 72, 96, and 120 h.

### 2.4 Cytokine detection assay

Plasma from the patients with NPC was isolated within 2 h after blood collection and frozen at −80 °C for later use. The Magnetic Luminex Performance assay (R&D) was applied to evaluate TNF‐a, VEGF, GM‐CSF, IL‐12p70, IL‐10, IL‐8, IL‐6, IL‐4, IL‐2, and IL‐1β. We used the ELISA Kit purchased from the R&D company to determine plasma TGF‐ β1 level. All detection assays were performed according to the manufacturer's protocols.

### 2.5 Statistical analysis

All statistical analyses were performed using GraphPad Prism 8.0. Differences between Healthy donors and patients with NPC were assessed using an unpaired *t*‐test. Wilcoxon matched‐pairs signed‐rank test was used in comparing the differences between blood samples collected on different days. A *P*‐value <0.05 was considered statistically significant. Data are shown as the mean ± SEM.

## 3 RESULTS

### 3.1 Patients characteristics

From February 25th to May 25th, samples from 96 NPC patients and 10 healthy donors were collected. Among the 96 NPC patients, 81 (84.4%) were diagnosed with locoregionally advanced disease (stage III/IVA). GP induction chemotherapy was administered in 55 (57.3%) patients, among whom 39 patients have blood samples collected on day 0, day 8, and day 21 (Table S1, Figure S1A).

### 3.2 Immunosuppressive myeloid cells

**FIGURE 1:**
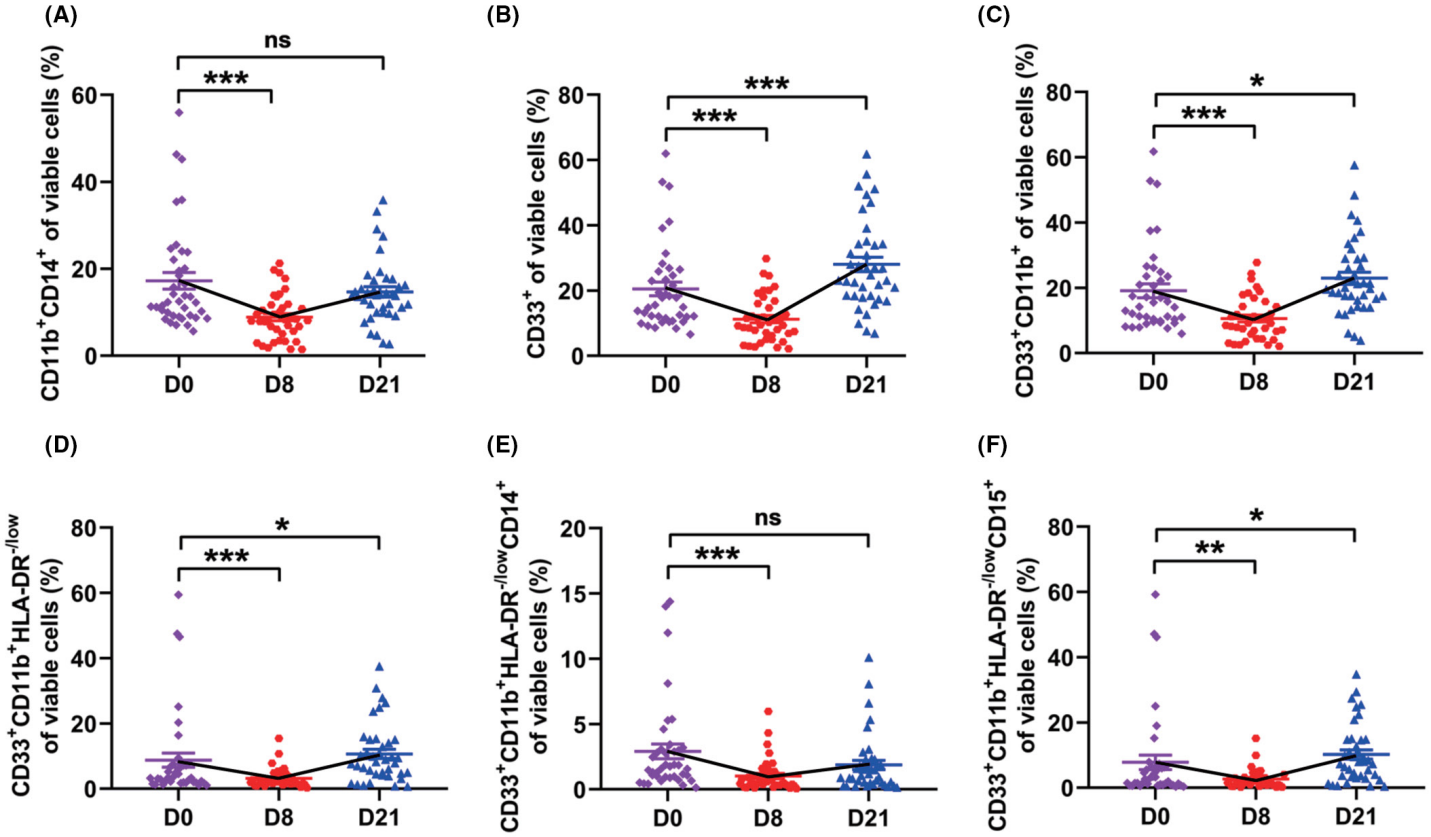
The influence of gemcitabine and cisplatin chemotherapy (GP) on myeloid cells. The dynamic changes of CD11b+CD14+ monocytes (A), CD33+ common myeloid cells (B), CD33+CD11b+ myeloid cells (C), myeloid‐derived suppressor cells (CD33+CD11b+HLA‐DR−/low, MDSCs) (D), CD14+ monocytic MDSCs (E), and CD15+ granulocytic MDSCs (F) in 39 patients during GP treatment. (*P <0.05, **P <0.01, ***P <0.001, ns: not statistically significant)

The levels of immunosuppressive myeloid cells, including immature CD11b+CD14+ monocytes, CD33+ myeloid cells, CD33+CD11+ myeloid cells, total MDSCs (CD33+CD11+HLA‐DR−/low), CD14+ monocytic MDSCs, and CD15+ granulocytic MDSCs (Figure S2), was significantly higher in NPC patients compared to healthy donors. After administered GP therapy, the levels of these cells decreased significantly on day 8 (all *P* values <0.05). Moreover, monocytic MDSC was still at a low level on day 21 (Figure 1).

### 3.3 T cells

In NPC patients, the levels of regulatory T cell (Tregs, 4.0% vs. 2.3%) and terminal differentiated CD8+ T cell (50.2% vs. 36.0%) significantly increased. On the contrary, the levels of CD3+ T cells (54.6% vs. 71.7%), naïve CD4+ (32.6% vs. 47.1%), and naïve CD8+ (24.1% vs. 38.2%) T cells decreased. The levels of central memory CD4+ T cells (25.2% vs. 19.7%) were higher in NPC patients. The level of central memory CD8+ T cells, effector memory CD4+ T cells, and effector memory CD8+ T cells were similar between NPC patients and healthy donors (Figure S3 and S4).

**FIGURE 2:**
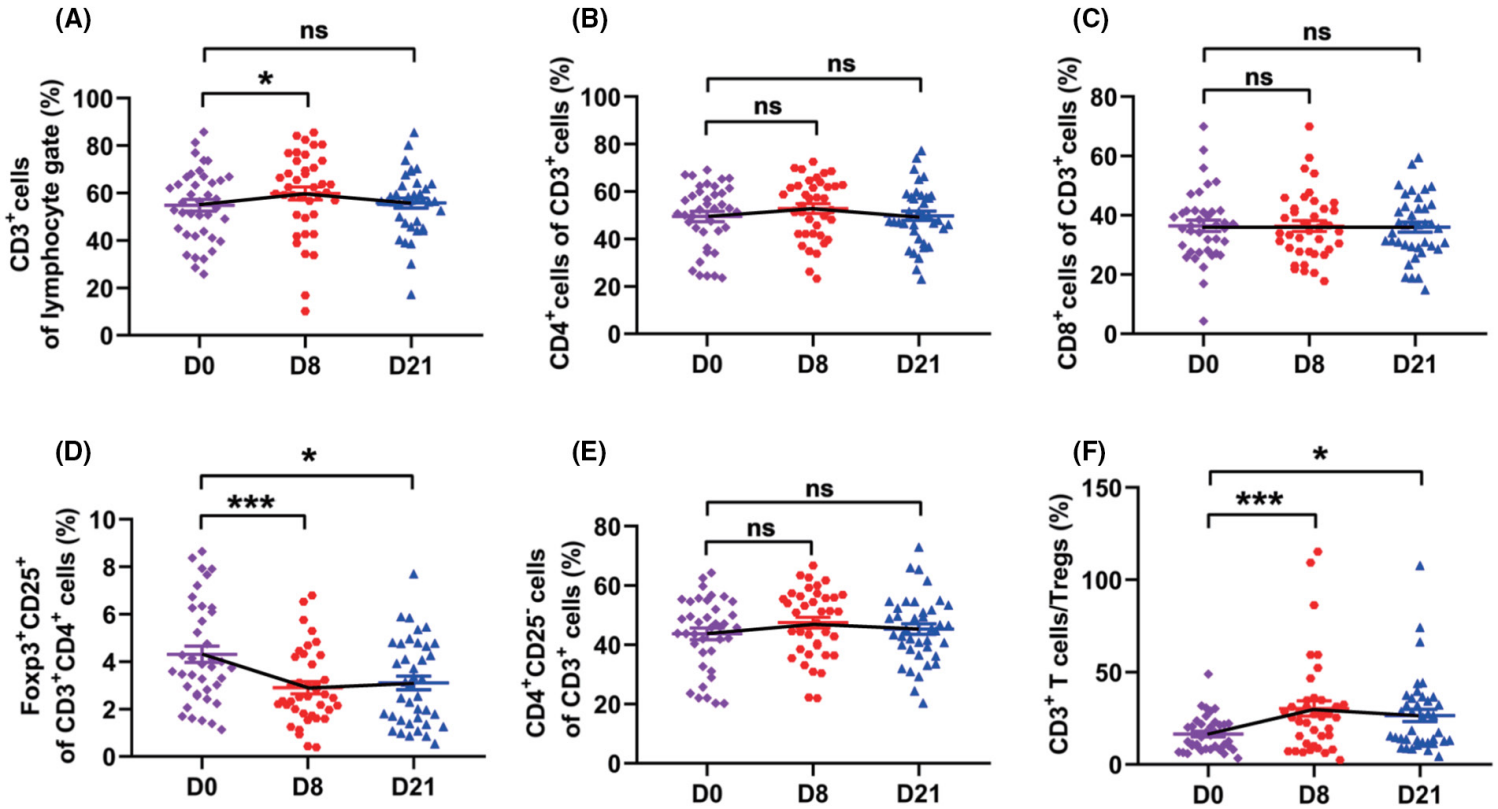
The effect of GP on T‐cell subsets. The dynamic changes of CD3+ cells (A), CD4+ T cell (B), CD8+ T cells (C), regulatory T cells (CD3+CD4+CD25+Foxp3+, Tregs) (D), conventional T cells (CD4+CD25−) (E), CD3+/Tregs ratios (F) in 39 patients during GP treatment. (*P <0.05, ***P <0.001, ns: not statistically significant)

**FIGURE 3:**
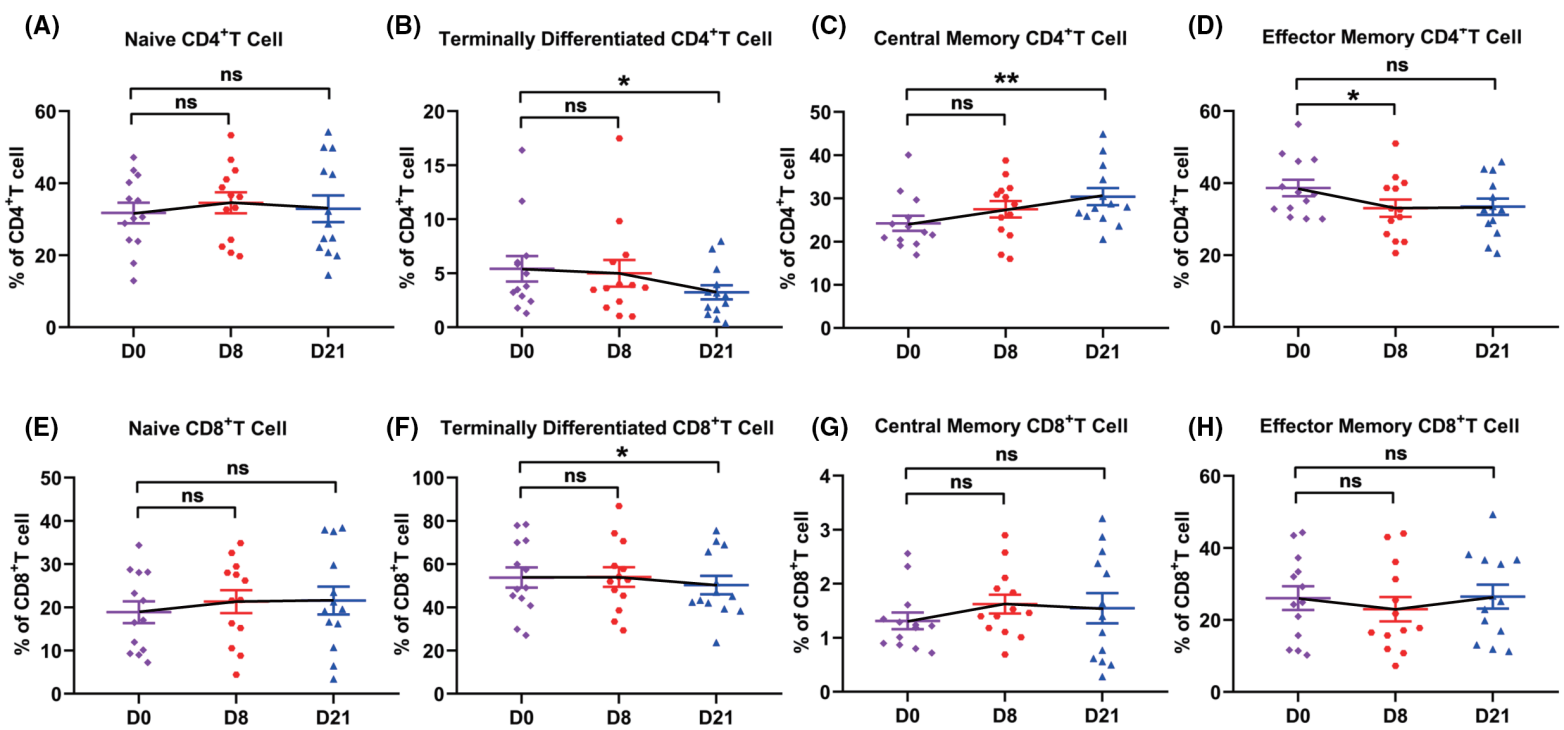
The effect of GP treatment on CD4+/CD8+ T‐cell subtypes. The dynamic changes of CD3+CD4+CD45RA+CCR7+naïve CD4+ T cells (A), CD3+CD4+CD45RA+CCR7− terminally differentiated CD4+ T cells (B), CD3+CD4+CD45RA‐CCR7+ central memory CD4+ T cells (C), CD3+CD4+CD45RA‐CCR7− effector memory CD4+ T cells (D), CD3+CD8+CD45RA+CCR7+ naïve CD8+ T cells (E), CD3+CD8+CD45RA+CCR7− terminally differentiated CD8+ T cells (F), CD3+CD8+CD45RA‐CCR7+ central memory CD8+ T cells (G), and CD3+CD8+CD45RA‐CCR7− effector memory CD8+ T cells (H) in 13 NPC patients during GP treatment. (*P <0.05, ns: not statistically significant)

**FIGURE 4:**
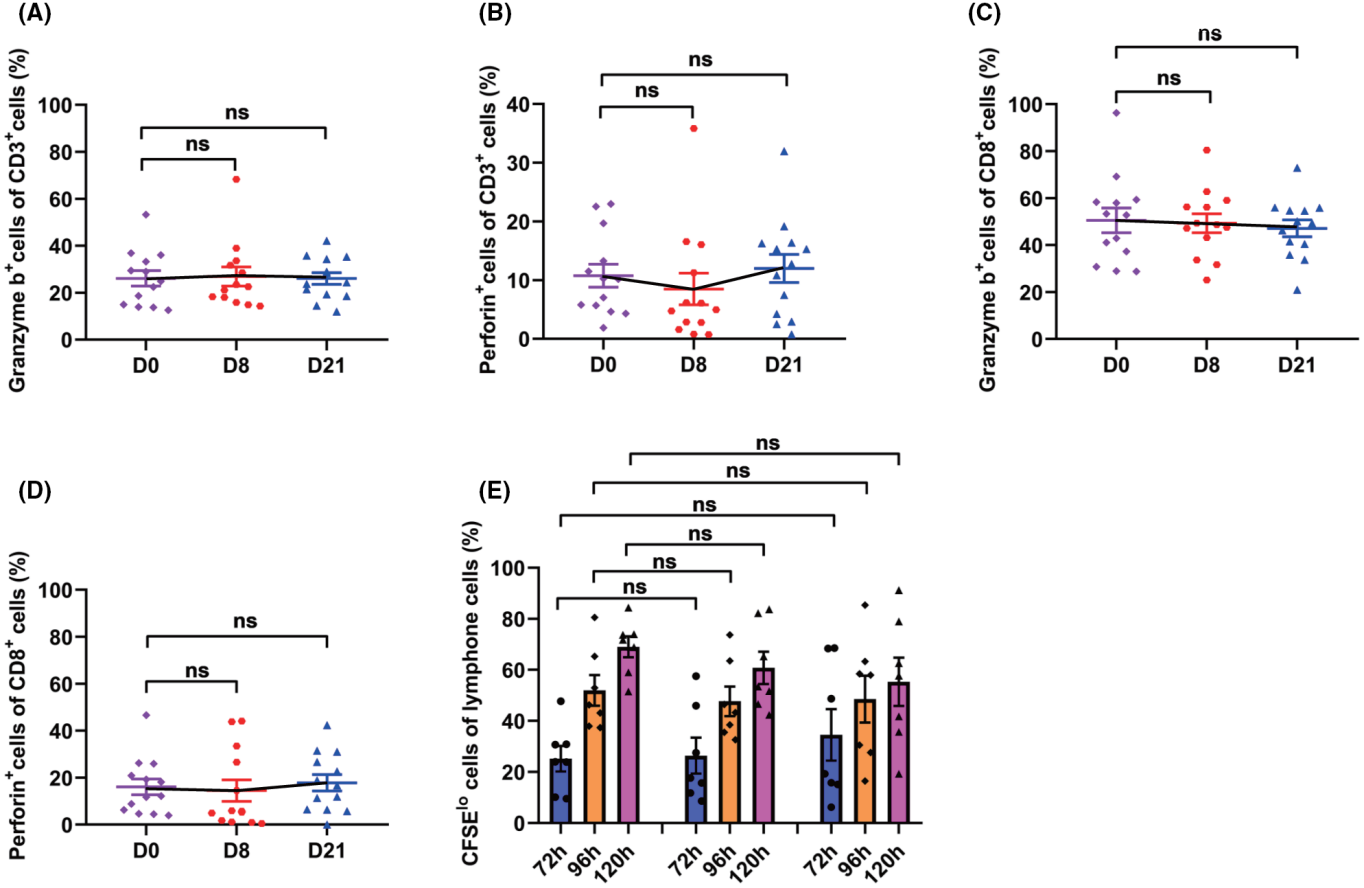
The influence of GP treatment on T‐cell function and lymphocyte proliferation. A‐D. The dynamic changes of granzyme B or perforin positive CD3+ T cells and CD8+ T cells in 13 NPC patients during GP treatment. E. The dynamic changes in the proliferative capacity of the lymphocytes of seven patients during GP treatment. (ns: not statistically significant)

After GP chemotherapy, the numbers of Tregs decreased significantly (from 4.3% to 2.9%) and still at a low level on day 21 (3.1%, all *P*‐value <0.05, Figure 2D); terminal differentiated CD4+ (5.42% vs. 3.24%) and CD8+ (53.8% vs. 50.3%) T cells, two exhausted‐like populations, also declined on day 21 (all *P*‐value <0.05, Figure 3B,F). Meanwhile, the level of CD3+ T cells (from 54.8% to 59.8%, *P* = 0.04, Figure 2A) increased on day 8, and central memory CD4 + T cells increased to an even higher level (from 54.8% to 59.8%, *P* = 0.04, Figure 3C). The frequencies of CD4+ and CD8+ T cells (Figure 2B,C), naïve CD4+ and naïve CD8+ T cells (Figure 3A,E), and effector memory CD4+ and CD8+ T cells (Figure 3D,H) remained stable during treatment. As for the function of the T cells, their cytotoxic and proliferative activities were not impaired after GP administration (all *P*‐value >0.05, Figure 4A–E).

### 3.4 B cells

**FIGURE 5:**
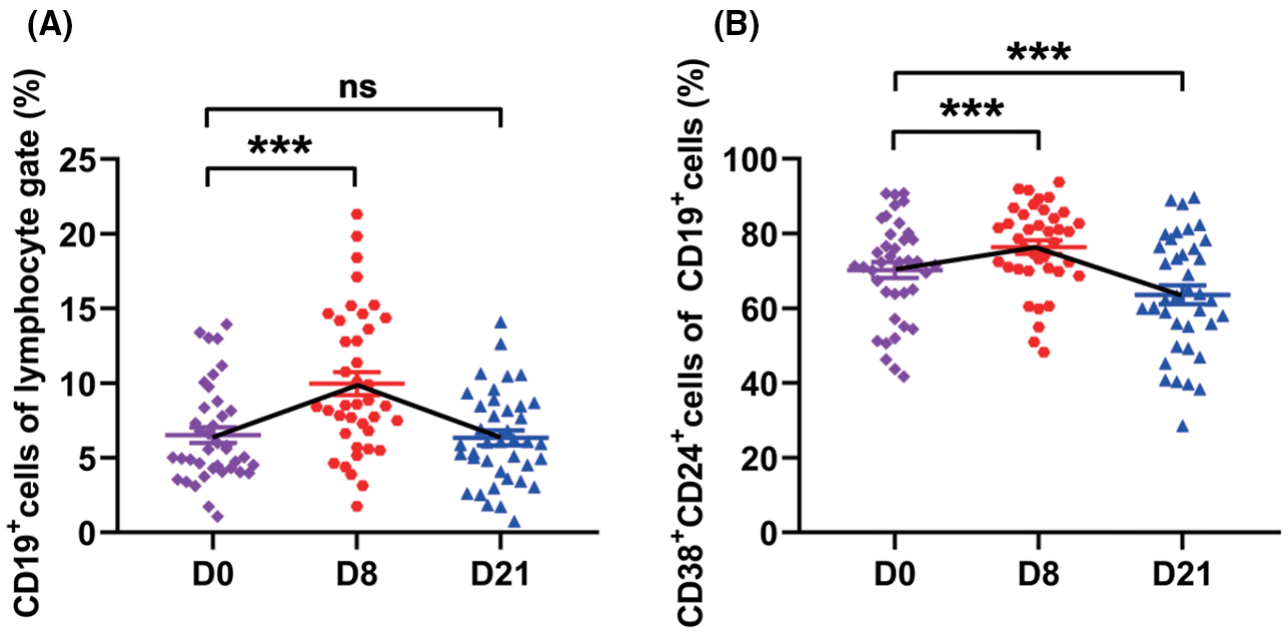
The effect of GP treatment on B cells. A‐B. The dynamic changes in the levels of CD19+ B cells and CD19+CD38+CD24+ regulatory B cells in 39 patients during GP treatment. (***P <0.001, ns: not statistically significant)

Patients with NPC have similar levels of total CD19+ B cells and regulatory B cells (CD19 + CD38 + CD24+, Bregs) with healthy donors. (Figure S6) After GP administration, the level of total B cells increased transiently on day 8 (from 6.5% to 10.0%, *P* <0.001, Figure 5A). Bregs lightly increased on day 8 (from 70.2% to 76.4%, *P* <0.001), but decreased to a lower level on day 21 (63.6%, Figure 5B).

### 3.5 Cytokine

**FIGURE 6:**
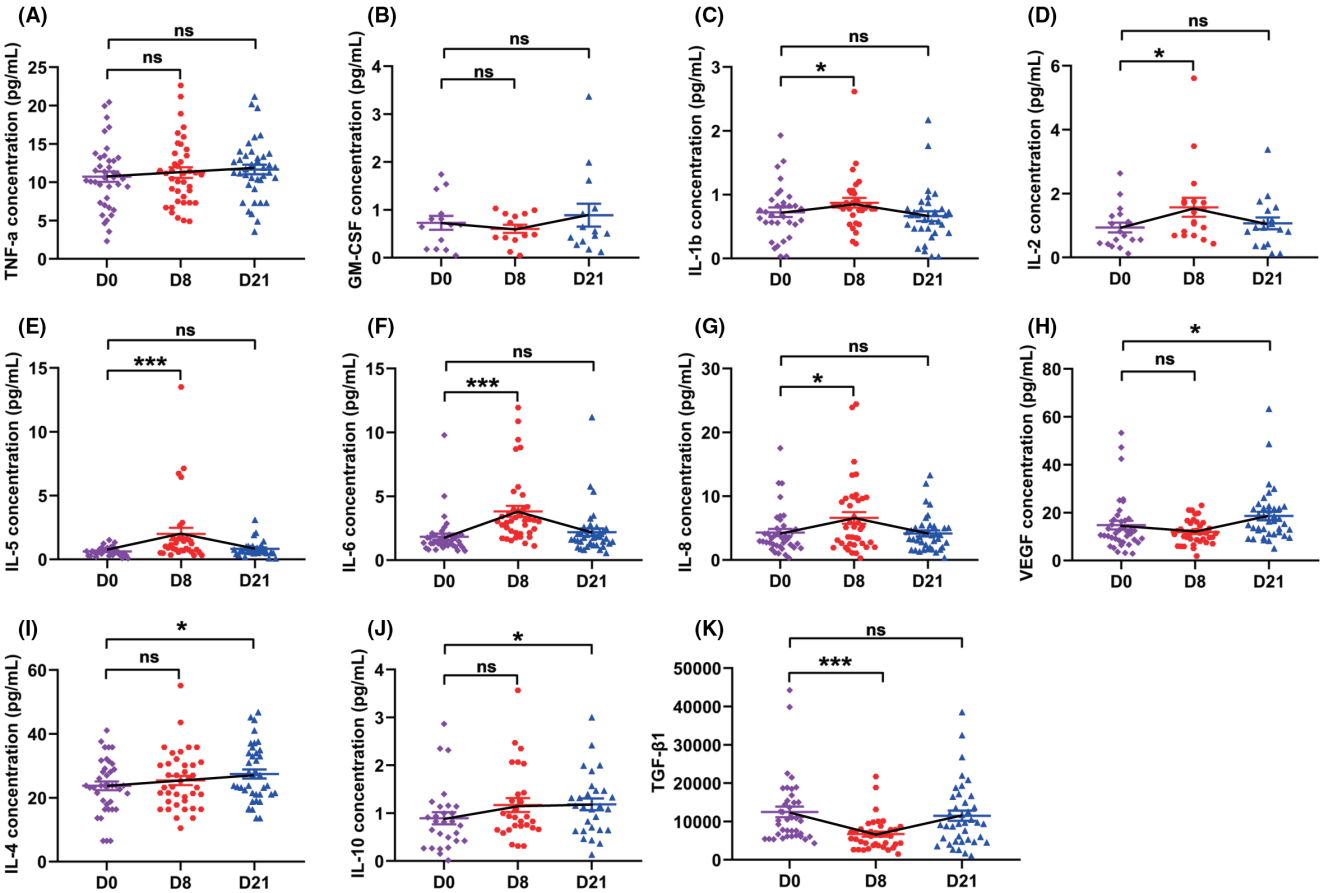
The effect of GP on immunomodulatory plasma proteins. The dynamic changes in the levels of TNFα, GM‐CSF, IL‐1b, IL‐2, IL‐5, IL‐6, IL‐8, VEGF, IL‐10, IL‐4, and TGF‐β1 (A‐K) in 39 patients during GP treatment. (*P <0.05, ***P <0.001, ns: not statistically significant)

We then examined the dynamic changes of plasma cytokine by using an 11‐cytokines detection assay and TGF‐β1 ELISA assays. The levels of TNF‐α and GM‐CSF were kept stable during treatment (Figure 6A,B). The levels of conventional pro‐inflammatory cytokines including IL‐1β, IL‐2, IL‐5, IL‐6, and IL‐8 significantly increased from day0 to day8 (Figure 6C–G). The concentrations of VEGF, IL‐4, and IL‐10 significantly increased on day21 (Figure 6H–J). By contrast, the concentration of anti‐inflammatory TGFβ‐1 was significantly decreased after GP administration but was restored after the resting period (Figure 6K). The levels of IL‐12P70 and IFNγ were lower than the detection limit of the kit used (data not shown).

## 4 DISCUSSION

To the best of our knowledge, this study first demonstrated the immune‐modulation effect of GP induction chemotherapy in patients with locoregionally advanced NPC. It could deplete the immunosuppressive cells such as immature monocytes, MDSCs, and regulatory lymphocytes, and potentially activated the antitumor immune response. This study might provide the basis for the combination of GP chemotherapy and immunotherapy in future studies.

MDSCs are heterogeneous populations of myeloid‐derived cells whose terminal differentiation and maturation stop at different stages, showing a strong suppressive effect on T cells.^10^ In NPC, the intratumoral MDSCs were differentiated from circulating CD33+ common myeloid cells and were expanded by cancer cell‐secreted GM‐CSF, IL‐1β, and IL‐6.^11^ These populations, not only accelerate the proliferation and invasion of NPC cells^12^ but also suppress the proliferation of antitumor lymphocytes such as CD4+ and CD8+ T cells. Therefore, the depletion of MDSCs might suppress tumor progression as well as activate antitumor immunity.^13^, ^14^ In the current study, we found that the increased MDSCs in NPC patients were dramatically decreased by GP induction chemotherapy. These findings were similar to the previous studies investigating gemcitabine in pancreatic carcinoma.^15^ MDSCs restored on day 21 might be because patients would regularly receive polyethylene glycol‐granulocyte colony‐stimulating factor after GP administration to prevent leukopenia. However, the transient decline of MDSCs induced by GP therapy still provided a valuable window for the combination of immunotherapy.

Regulatory lymphocytes, including Tregs and Bregs, are important immune‐suppressive populations. These two populations can suppress the cytotoxic and proliferative capacity of cytotoxic CD8+ T cells through immunosuppressive cytokines such as IL‐10 and TGF‐β1.^16^ Our results demonstrated that after GP administration, the level of Treg decreased significantly, which might be a result of both MDSC reduction^17^, ^18^ and the directly GP‐induced depletion.^19^ Bregs also significantly declined after GP administration. Since low levels of Tregs and Bregs were associated with a good response to immunotherapy, the immune modulation effect of GP might provide a good immune microenvironment for the combination of immunotherapy.

Both CD4+ and CD8+ T cells are the important executors of antitumor immune response.^20^, ^21^ Traditionally, these cells were thought to be vulnerable to chemotherapy.^22^ However, in the current studies, we found that after GP administration, the levels of these cells were not decreased; the levels of CD3+ T cells and central memory CD4+ T cells were even higher. The increment in naïve CD4+ and CD8+ T cells was also observed, although it was not statically significant. On the other hand, the level of terminal differentiated CD4+ and CD8+ T cells, an exhausted‐like population, was decreased after GP administration. As for the function of T cells, the cytotoxic activity of CD3+ and CD8+ T cells was not weakened after GP administration. All these findings indicated that the T cell‐mediated antitumor immune system was not impaired after GP administration. Under this premise, with GP depleting suppressive immune cells, the addition of immunotherapy could further activate the antitumor immune response.

This study has some limitations. First, we only examined three‐time points to investigate the change in circulating immune cells and plasma cytokines after GP administration. Second, the exact mechanism through which GP regulates the immune system needs further study. However, our study still provides a basis for the combination of GP therapy and immunotherapy. Besides, our recent trials show that adjuvant metronomic capecitabine is also effective in NPC.^23^ Metronomic chemotherapy works through antiangiogenesis, but it also has immune effects and can reduce Treg and MDSCs and cause dendritic cell maturation.^24^ The addition of immunotherapy to adjuvant metronomic chemotherapy should also be explored. Currently, we are conducting two prospective randomized clinical trials (NCT03984357 and NCT03619824) to investigate whether additional immunotherapy to GP induction chemotherapy could further improve survival in locoregionally advanced NPC.

## 5 CONCLUSION

Our results demonstrated the immune modulation effect of GP therapy in patients with NPC, which could offer a solid basis for its combination with immunotherapy in future studies.

## CONSENT FOR PUBLICATION

The manuscript was read and consent for publication by all authors.

## CONFLICT OF INTEREST

There is no competition of interest.

## AUTHOR CONTRIBUTIONS

Conception and design: Xiao‐Min Li, Xiao‐Min Zhang, Jun‐Yan Li, Ning Jiang, Na Liu, Yuan Zhang, and Jun Ma; Sample collection: Xiao‐Min Li, Jun‐Yan Li, Lei Chen, Ying‐Qin Li, Ling‐Long Tang, Guan‐Qun Zhou, Yan‐Ping Mao, and Wen‐Fei Li; Analysis: Xiao‐Min Li, Jun‐Yan Li, Ning Jiang, Yuan Zhang, and Jun Ma; Manuscript writing and revision: Xiao‐Min Li, Jun‐Yan Li, Ning Jiang, Na Liu, Yuan Zhang, and Jun Ma.

## Supporting information


Figure S1
Figure S2
Figure S3
Figure S4
Figure S5
Figure S6
Table S1
Table S2

## Data Availability

All the key raw research data was uploaded onto the Research Data Deposit public platform of Sun Yat‐sen University Cancer Center (http://www.researchdata.org.cn) and could be obtained upon reasonable request.
