# Supplementary material for: The immune modulation effects of gemcitabine plus cisplatin induction chemotherapy in nasopharyngeal carcinoma
Source: Cancer Med. 2022 Mar 30;11(18):3437–44. doi: 10.1002/cam4.4705 (PMC9487869; doi:10.1002/cam4.4705)
Supplement: Supplementary file 1 — Figure S1 Figure S2 Figure S3 Figure S4 Figure S5 Figure S6 Table S1 Table S2 [file CAM4-11-3437-s001.docx]

**
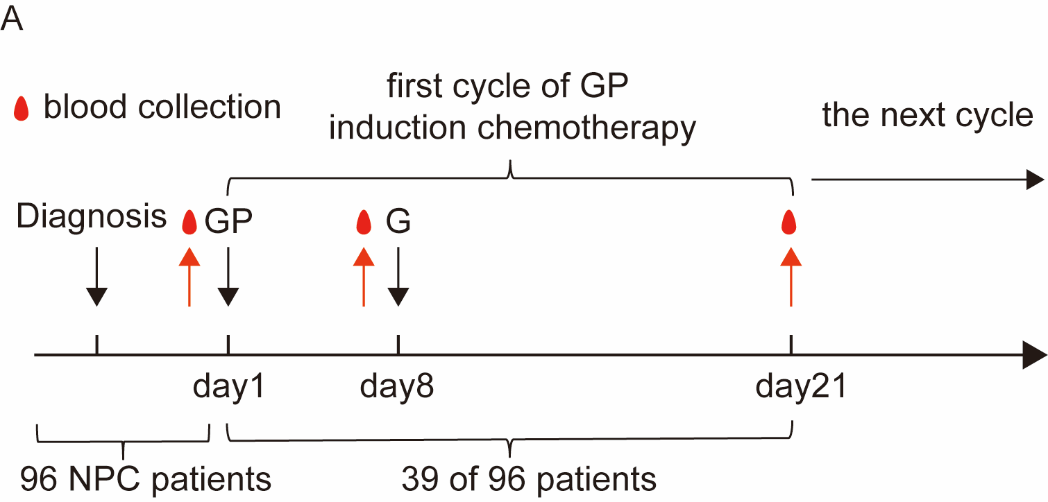
**

**Supplementary Figure 1.** The study outlines. Blood samples were collected from 96 newly diagnosed patients before treatment (day0). 39 of them provided three blood samples during their first cycle of GP treatment (day0: 1 day before the first GP treatment, day8: the day of gemcitabine monotherapy, and day21: the last day of the GP cycle).

**
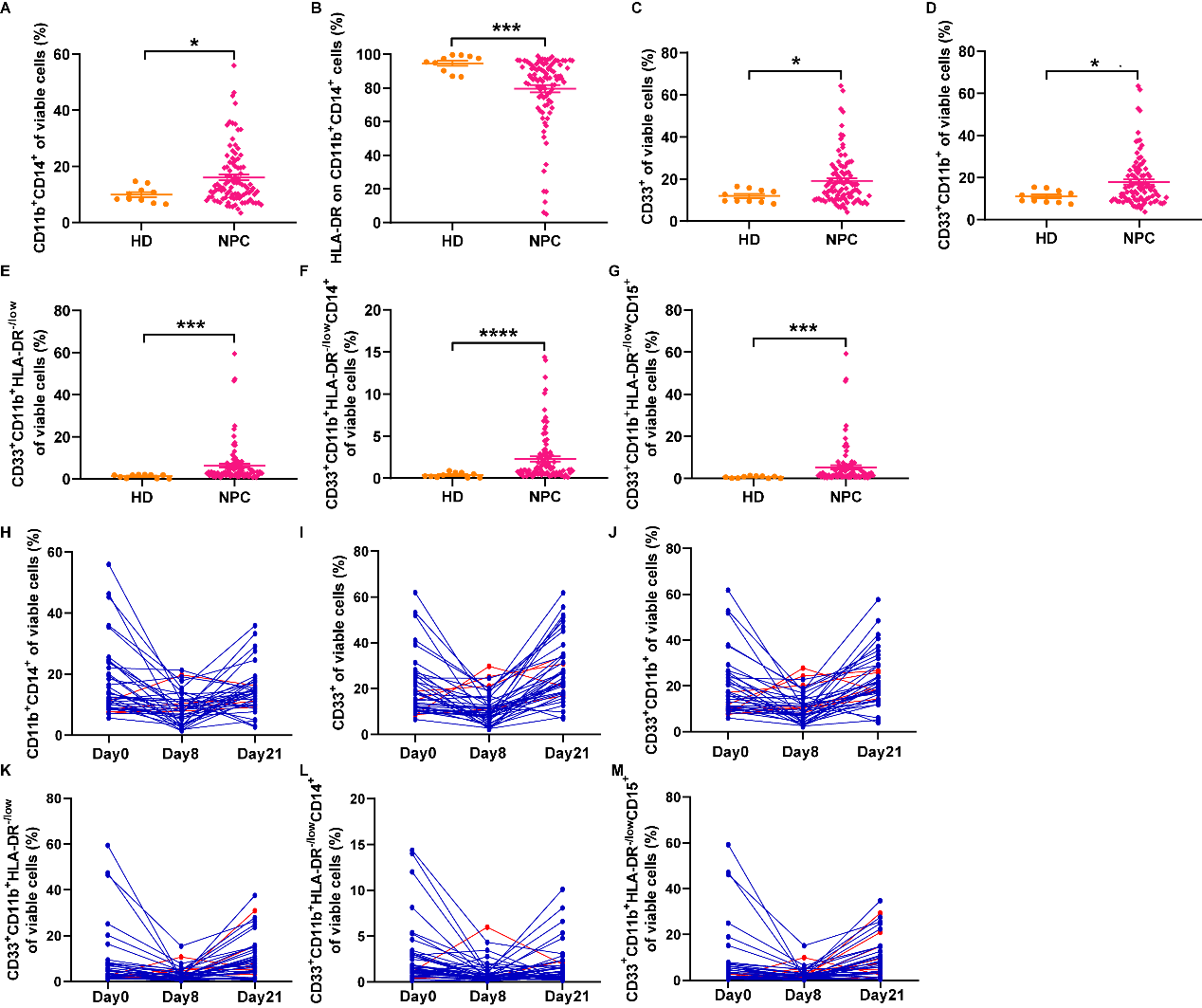
**

**Supplementary Figure 2.** The levels of CD11b+CD14+ monocytes (**A**), CD33+ common myeloid cells (**B**), CD33+CD11b+ myeloid cells (**C**), CD33+CD11b+HLA-DR-/low MDSCs (**D**), CD33+CD11b+HLA-DR-/lowCD14+ monocytic MDSCs (**E**), and CD33+CD11b+HLA-DR-/lowCD15+ granulocytic MDSCs (**F**) in 10 healthy donors and 96 patients with NPC. The differences were analyzed using an unpaired t-test with the Mann-Whitney test (**P* < 0.05, ***P* < 0.01, ****P* < 0.001, ns: not statistically significant).


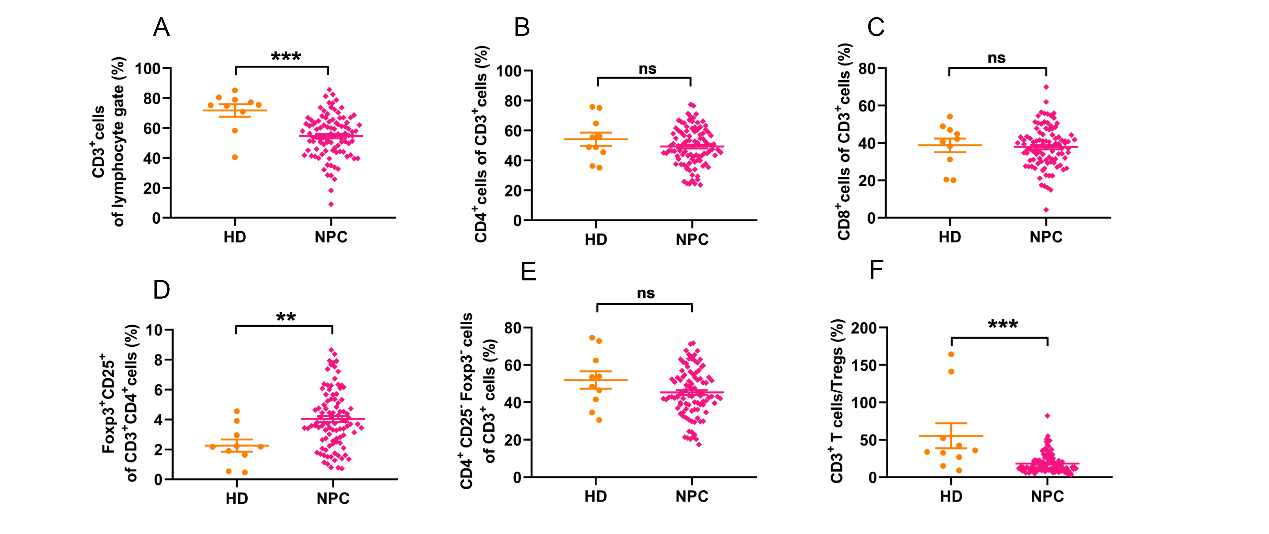


**Supplementary Figure 3.** The levels of circulating CD3+ cells (**A**), total CD4+ T cell (**B**), CD8+ T cells (**C**) CD3+CD4+CD25+Foxp3+Tregs (**D**), CD4+CD25- conventional T cells (**E**), CD3+/Tregs ratios (**F**) in 10 healthy donors and 96 patients with NPC. The differences were analyzed using an unpaired t-test with the Mann-Whitney test (**P* < 0.05, ***P* < 0.01, ****P* < 0.001, ns: not statistically significant).


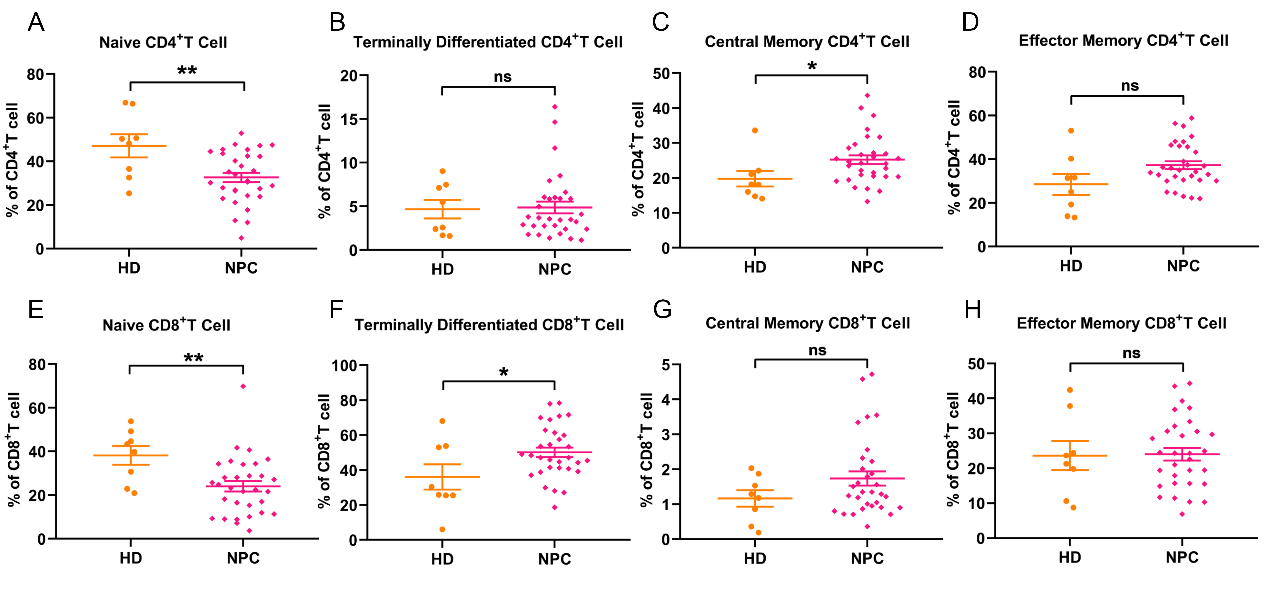


**Supplementary Figure 4.** **A-H**. The levels of naïve CD4+ T cells (CD3+CD4+CD45RA+CCR7+) (**A**), terminally differentiated CD4+ T cells (CD3+CD4+CD45RA+CCR7-) (**B**), central memory CD4+ T cells (CD3+CD4+CD45RA-CCR7+) (**C**), effector memory CD4+ T cells (CD3+CD4+CD45RA-CCR7-) (**D**), naïve CD8+ T cells (CD3+CD8+CD45RA+CCR7+) (**E**), terminally differentiated CD8+ T cells (CD3+CD8+CD45RA+CCR7-) (**F**), central memory CD8+ T cells (CD3+CD8+CD45RA-CCR7+) (**G**), and effector memory CD8+ T cells (CD3+CD8+CD45RA-CCR7-) (**H**) in 8 healthy donors and 31 NPC patients. The differences were analyzed using an unpaired t-test with the Mann-Whitney test (**P* < 0.05, ***P* < 0.01, ****P* < 0.001, ns: not statistically significant).


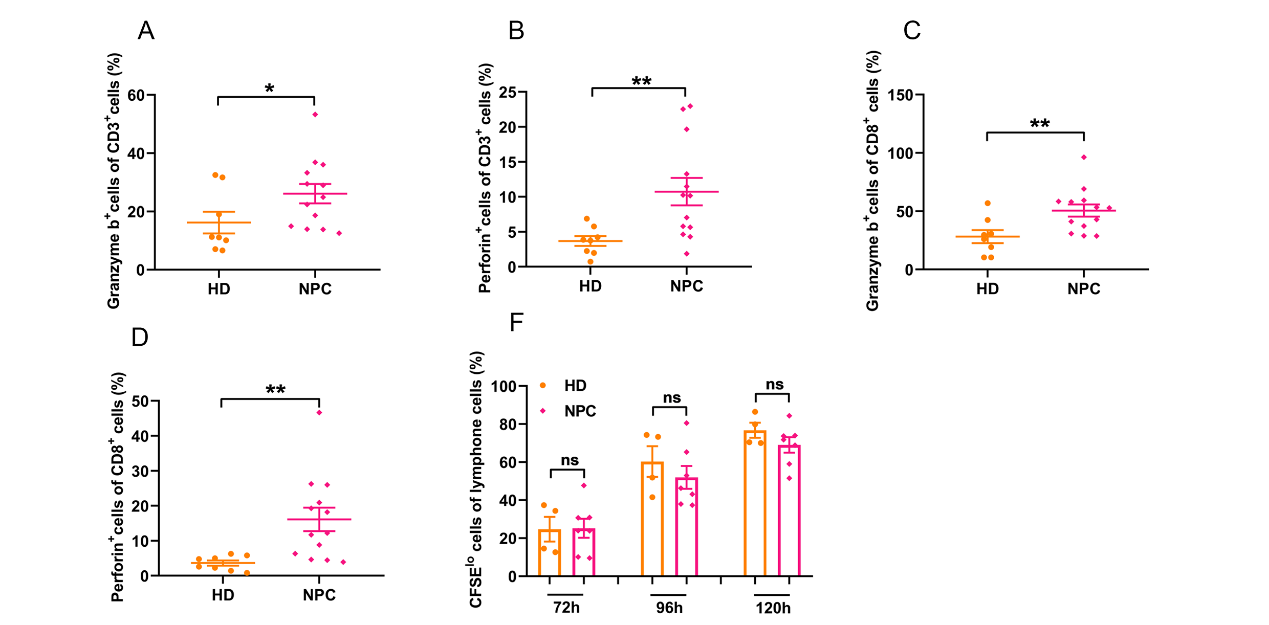


**Supplementary Figure 5.** The comparison of levels of granzyme B or perforin positive CD3+ T cells (**A** and **B**) and CD8+ T cells (**C** and **D**) i in 8 healthy donors and 13 NPC patients. The differences were analyzed using an unpaired t-test with the Mann-Whitney test (**P* < 0.05, ***P* < 0.01, ****P* < 0.001, ns: not statistically significant).

**
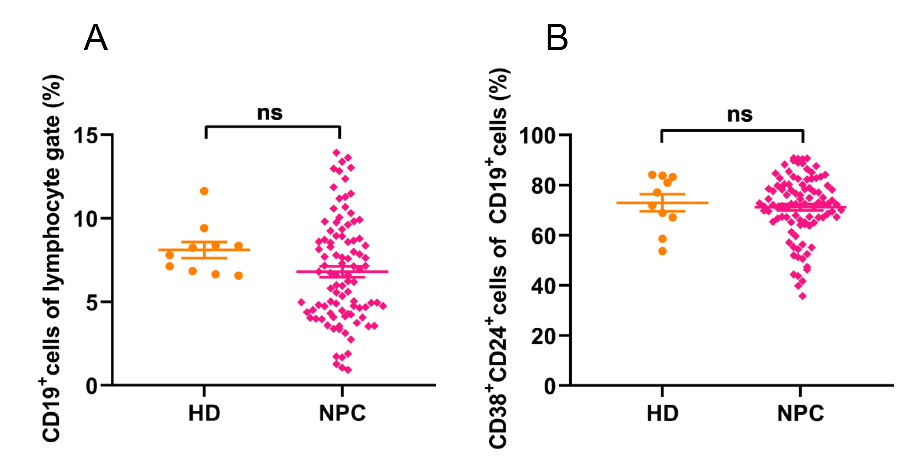
**

**Supplementary Figure 6**. The comparison of levels of CD19+ B cells (**A**) and CD19+CD38+CD24+ regulatory B cells (**B**) between 10 healthy donors and 96 patients with NPC. The differences were analyzed using an unpaired t-test with the Mann-Whitney test (**P* < 0.05, ***P* < 0.01, ****P* < 0.001, ns: not statistically significant).

**Supplementary Table 1**. Characteristics of patients with nasopharyngeal carcinoma (*N* = 96).

| Patients characteristics | All patients (%) | Tracked cohort ^c^ (%) |
| --- | --- | --- |
| Age |  |  |
| Median | 47 | 48 |
| Range | 17–80 | 17–65 |
| Gender |  |  |
| Male | 76 (79.2) | 34 (87.2) |
| Female | 20 (20.8) | 5 (12.8) |
| T category ^a^ |  |  |
| T1 | 5 (5.2) | 0 (0.0) |
| T2 | 7 (7.3) | 1 (2.6) |
| T3 | 49 (51.0) | 21 (53.8) |
| T4 | 35 (36.5) | 17 (43.6) |
| N category ^a^ |  |  |
| N0 | 6 (6.3) | 4 (10.3) |
| N1 | 44 (45.8) | 10 (25.6) |
| N2 | 32 (33.3) | 19 (48.7) |
| N3 | 14 (14.6) | 6 (15.4) |
| M category ^a^ |  |  |
| M0 | 88 (91.7) | 36 (92.3) |
| M1 | 8 (8.3) | 3 (7.7) |
| Clinical stage ^a^ |  |  |
| I | 1 (1.0) | 0 (0.0) |
| II | 7 (7.3) | 1 (2.6) |
| III | 43 (44.8) | 17 (43.6) |
| IVa | 38 (39.6 ) | 18 (46.1) |
| IVb | 7 (7.2) | 3 (7.7) |
| Treatment  Induction chemotherapy  GP regimen ^b^  Other regimens |  |  |
|  | 73 (76.0) | 39 (100.0) |
|  | 55 (57.3) | 39 (100.0) |
|  | 18 (18.6) | 0 (0.0) |
| Concurrent chemo-radiotherapy  Radiotherapy alone | 21 (21.9) | 0 (0.0) |
|  | 2 (2.1) | 0 (0.0) |
| Total | 96 (100.0) | 39 (100.0) |

^a^ According to the 8th American Joint Committee on Cancer/Union for International Cancer Control (AJCC/UICC) of the staging system.

^b^ Gemcitabine and platinum chemotherapy

^c^ Peripheral blood was collected from patients at three timepoints (on Day 0, Day 8, and Day 21, respectively, of the first cycle of inductive gemcitabine and platinum chemotherapy)

**Supplementary Table 2.** Antibodies and gating strategy in flow cytometry analysis.

| Markers | Fluorescence | Clone | Supplier |
| --- | --- | --- | --- |
| CD3 | FITC | HIT3a | Biolegend |
| CD4 | PerCP/CY-5.5 | A161A1 | Biolegend |
| CD4 | APC/CY7 | RPA-T4 | Biolegend |
| CD8 | PerCP | SK1 | Biolegend |
| CD8 | Alexa Fluor 700 | SK1 | Biolegend |
| CD11b | Alexa Fluor700 | ICRF44 | Biolegend |
| CD14 | FITC | HCD14 | Biolegend |
| CD15 | PE | HI98 | Biolegend |
| CD16 | Brilliant Violet 421 | 3G8 | Biolegend |
| CD19 | FITC | HIB19 | Biolegend |
| CD19 | PerCP/CY5.5 | HIB19 | Biolegend |
| CD24 | APC | ML5 | Biolegend |
| CD25 | APC | BC96 | Biolegend |
| CD33 | Brilliant Violet 421 | WM53 | Biolegend |
| CD38 | PE | HB-7 | Biolegend |
| CD45RA | Brilliant Violet510 | HI100 | Biolegend |
| CD56 | PE/CY7 | NCAM | Biolegend |
| CCR7 | Brilliant Violet421 | G043H7 | Biolegend |
| FOXP3 | PE | 206D | Biolegend |
| Granzyme B | APC | QA16A02 | Biolegend |
| HLA-DR | PE/CY7 | L243 | Biolegend |
| Perforin | Brilliant Violet421 | dG9 | Biolegend |
| mouse IgG1κ | APC | MOPC-21 | Biolegend |
| mouse IgG1κ | PE | ICFC | Biolegend |
